# Ancillary polymorphic floral traits between two morphs adaptive to hawkmoth pollination in distylous plant *Tirpitzia sinensis* (Linaceae)

**DOI:** 10.1186/s12870-022-03659-w

**Published:** 2022-06-03

**Authors:** Xiaoyue Wang, Demei Hu, Yan Chen, Mengda Xiang, Hanqing Tang, Yin Yi, Xiaoxin Tang

**Affiliations:** 1 Key Laboratory of State Forestry Administration On Biodiversity Conservation in Karst Mountainous Areas of Southwestern China, Guizhou Normal University, Guiyang, 550025 China; 2 School of Life Sciences, Guizhou Normal University, Guiyang, 550025 China

**Keywords:** Distyly, Nectar traits, Pollinator, Pollination efficiency, Breeding system

## Abstract

**Background:**

Floral morphs are characterized differentiations in reciprocal positions of sexual organs and ancillary floral traits in heterostylous plants. However, it remains unclear how differential floral morphs ensure reproductive success between morphs using the same pollinator.

**Results:**

Measurements of floral traits in white-flowered *Tirpitzia sinensis* with long corolla tubes indicated that it is typically distylous, long-styled (L-) morph producing more but smaller pollen grains per flower than short-styled (S-) morph. Both morphs secreted more nectar volume at night than in the day and the sugar composition was rich in sucrose, potentially adaptive to pollination by hawkmoths (*Macroglossum* spp.) which were active at dusk. A bumblebee species functioned as the nectar robber in both morphs and a honeybee as the pollen feeder in the S-morph. The L-morph secreted more nectar volume but relatively lower sucrose/hexose ratio than the S-morph. Floral visitation rate by hawkmoths was higher but its pollination efficiency was lower in the S-morph than the L-morph. Hand pollination treatments indicated self-incompatibility in *T. sinensis* and seed set of open-pollinated flowers did not differ between morphs.

**Conclusions:**

Our findings suggest that the two morphs differ with respect to traits relevant to pollination. The L-morph, with its exserted stigma, has more pollen grains per anther and a greater volume of nectar, which may prolong the foraging time and increase the pollination efficiency of hawkmoths. The S-morph has a higher sucrose/hexose ratio in its nectar which can be more attractive to hawkmoths and increase the visit rates. Ancillary polymorphic floral traits between two morphs are adaptive to hawkmoth and ensure reproductive success in distylous plant *T. sinensis*.

## Background

Heterostyly, including distyly and tristyly, exhibits variation and complexity in the positions of floral sexual organs [1–3]. Distyly is recorded from at least 28 angiosperm families, such as Primulaceae, Boraginaceae, Plumbaginaceae, Polemoniaceae, Polygonaceae and Linaceae, and accounts for 1–2% of flowering plant species [4]. Heterostyly is regarded as an adaptation avoiding self-pollination, promoting cross-pollination and reducing interference between male and female functions in the same flower [1, 3, 5].

Heterostyly usually occurs in narrow tubular flowers but rarely in open bowl-shaped flowers [3, 6]. In addition to reciprocal herkogamy, heterostyly may involve stamen characters (such as the number and size of pollen grains, the ornamentation of the pollen exine, and anther size and colour) and stigma depth and receptive area, features generally referred to as ancillary polymorphism [7–9]. Long-styled morphs (hereafter L-morphs, with short anthers) usually produce more but smaller pollen grains than short-styled morphs (S-morphs, with high anthers) [7, 8, 10]. The stigma of L-morphs is usually larger than that of S-morphs [11–13]. Distylous plants are usually self-incompatible [7, 14].

Nectar is generally present and concealed at the base of floral tubes in distylous plants. Long-tongued pollinators probe into the floral tube to seek nectar, thus transferring pollen [3, 6]. The differences in nectar traits (nectar volume, sugar concentration and nectar composition) could affect the pollinator’s behaviour [15]. It remains unclear how different morphs of distylous plants ensure reproductive success between morphs using the same pollinator. Whether the floral characters, especially nectar traits are related to it.

**Fig. 1:**
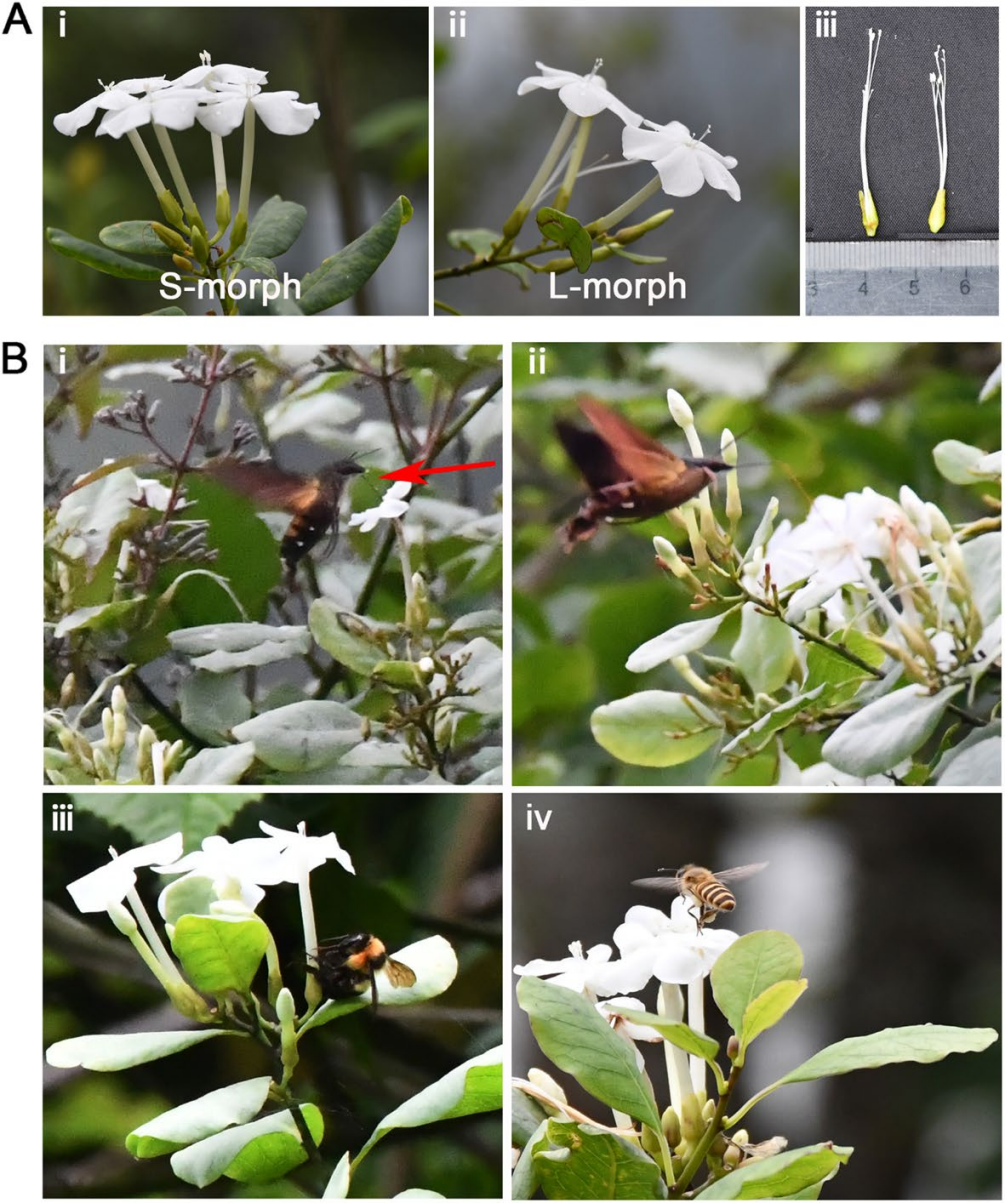
The short-styled (hereafter S-morph) and long-styled morphs (hereafter L-morph) of distylous *Tirpitzia sinensis* (A), and the main visitors of *T. sinensis* (B). **A** (i) the S-morph and (ii) L- morph of *Tirpitzia sinensis.* (iii) Pistil and stamen of L- and S- morphs. **B** (i, ii) Hawkmoth pollinator *Macroglossum* probed for the nectar secreted at the base of the long floral tube of *T. sinensis* (Note the pollen deposited on the tongue in i, marked with a red arrow). (iii) Bumblebees (*Bombus*) always robbed the nectar. (iv) Honeybees (*Apis*) mainly groomed the *T. sinensis* pollen into their corbiculae, acting as pollen thieves when visiting the flowers. All the photos are taken by Xiaoyue Wang and Demei Hu

*Tirpitzia sinensis* (Linaceae) is a distylous shrub or small tree 1–5 m tall with elliptic, obovate-elliptic or ovate leaves and scentless narrow white tubular flowers. It is widely distributed mainly in Guangxi, Guizhou, Yunnan, southeast China at an elevation of 300–2000 m, and usually grows in calcareous soil on mountain slopes or along trails. The 4 cm cymose inflorescence is generally terminal or axillary at the top of a stem or branch. Each flower consists of five green sepals and five white petals forming a floral tube. Nectar is usually present, concealed at the base of the floral tube. Five stamens surround the central four pistils. Flowers are homogamous and usually last 3–4 days. Plants usually flower from May to August. Capsules contain three to eight seeds and mature 3 months after fertilisation [16]. Our field investigation of pollination ecology revealed that *T. sinensis* is a typical distylous plant with L-morphs (anthers are low in the corolla, and stigmas are high, on long styles) and S-morphs (anthers are high, and stigmas are low) (Fig. 1A. i, ii, iii) and is widely distributed in the field. There is no significant difference in the numbers of L- and S-morphs of *T. sinensis* in field populations [17]. It is an ideal material with which to explore different adaptations of the L- and S-morphs of distylous plants to pollinators. Hawkmoths are important pollinators with exceptionally long tongues. They fly at dusk and feed from flowers while hovering [18, 19]. Hawkmoth pollination has been reported in a wide range of angiosperm taxa [20, 21]. It remains unclear whether hawkmoths could effectively pollinate *T. sinensis.*

This study aimed to (1) compare floral characters, especially the volume, sugar concentration and composition of nectar, between L- and S-morphs; (2) identify the effective pollinators of *T. sinensis* and compare the visit rate and pollination efficiency (pollen removal and receipt per visit) between L- and S-morphs; and (3) test whether *T. sinensis* is self-incompatible and compare natural seed production between L- and S-morphs.

## Results

### Differences in floral characters between the two morphs

Leaves of the S-morph were relatively larger than those of the L-morph (a marginally significant difference, see Table 1). The S-morph had larger sepals, wider blades, deeper floral tubes, longer stamens and larger pollen grains than the L-morph (all *P* < 0.05). The corolla length, blade length, pistil length, anther length, pollen grain number and pollen/ovule ratio of the L-morph were greater than those of the S-morph (all *P* < 0.05). Other floral traits including corolla width, opening diameter, tube diameter, anther width, anther thickness, duration of flowering of each flower (flower lifetime) and ovule number were not significantly different between the two morphs (all *P* > 0.05) (Table 1). Generally, the L-morphs had a longer pistil and produced more but smaller pollen grains per flower and consequently had a higher pollen/ovule ratio than the S-morph. Meanwhile, the S-morph had longer stamens, and produced fewer but larger pollen grains than the L-morph.

**Table 1:** Comparisons of vegetative and reproductive traits (mean ± SE) between long-styled morphs (hereafter L-morph) and short-styled (hereafter S-morph) morphs of *Tirpitzia sinensis* tested by a generalized linear model (GLM) analysis. Values of one morph significantly larger than the other are written in bold

Traits	L-morph	S-morph	Wald χ^2^	*P*
Leaf length (mm)	45.98 ± 0.57	**49.45 ± 0.96**	3.89	0.049
Leaf width (mm)	26.67 ± 0.57	**28.25 ± 0.55**	3.981	0.046
Sepal length (mm)	6.32 ± 0.12	**7.03 ± 0.12**	17.836	< 0.001
Sepal width (mm)	2.87 ± 0.04	**3.02 ± 0.04**	7.045	0.008
Corolla length (mm)	**23.64 ± 0.28**	22.49 ± 0.28	8.482	0.004
Corolla width (mm)	22.29 ± 0.27	21.63 ± 0.27	2.934	0.087
Blade length (mm)	**11.62 ± 0.14**	11.23 ± 0.14	4.056	0.044
Blade width (mm)	9.11 ± 0.15	**9.77 ± 0.16**	9.455	0.002
Opening diameter (mm)	1.49 ± 0.03	1.47 ± 0.02	0.368	0.544
Tube depth (mm)	32.19 ± 0.26	**35.05 ± 0.29**	54.607	< 0.001
Tube diameter (mm)	1.86 ± 0.03	1.87 ± 0.02	0.098	0.754
Pistil length (mm)	**36.26 ± 0.29**	30.64 ± 0.33	164.13	< 0.001
Stamen length (mm)	29.94 ± 0.26	**36.98 ± 0.29**	334.767	< 0.001
Anther length (mm)	**1.81 ± 0.06**	1.31 ± 0.03	50.254	< 0.001
Anther width (mm)	0.56 ± 0.02	0.53 ± 0.02	2.517	0.113
Anther thickness (mm)	0.36 ± 0.01	0.35 ± 0.01	0.071	0.79
Flower lifetime (days)	3.4 ± 0.07	3.5 ± 0.11	1.124	0.289
Pollen grain number	**3187.9 ± 120.8**	1268.2 ± 51.3	70.485	< 0.001
Pollen polar axis length (µm)	46.02 ± 0.62	**69.04 ± 0.85**	483.332	< 0.001
Pollen equatorial axis length (µm)	45.87 ± 0.56	**68.86 ± 0.93**	445.653	< 0.001
Ovule number	8.2 ± 0.1	8.2 ± 0.1	0.026	0.871
Pollen/ovule ratio	**394.42 ± 17.47**	154.41 ± 6.44	183.97	< 0.001

### Measurement of nectar volume, sugar concentration and properties

**Fig. 2:**
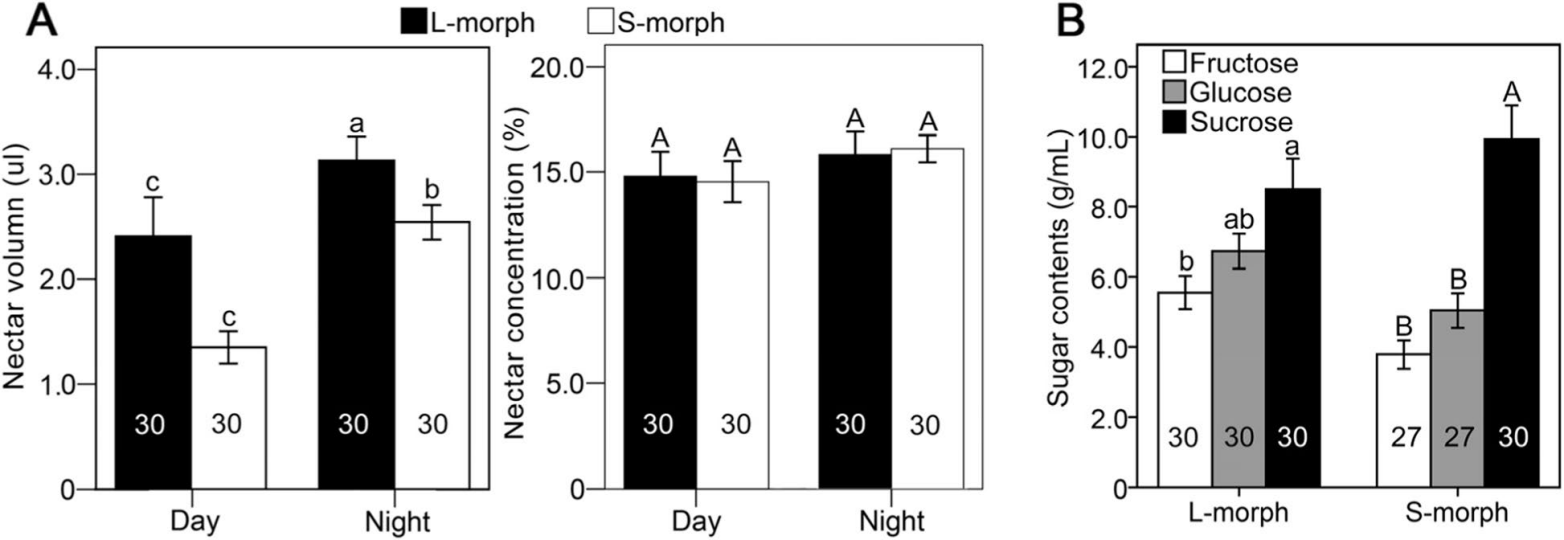
Comparison of nectar volume, sugar concentration (mean ± SE) of L- and S-morphs between day and night in *T. sinensis* (**A**) and comparison of fructose, glucose and sucrose composition (mean ± SE) in the L- and S-morph nectar of *T. sinensis* (**B**). Bars that share the same letters are not significantly different between treatments. Numbers in the bar graph represent the sample size

*T. sinensis* produced a significantly (*P* < 0.001, Wald χ^2^ = 47.197, df = 1) larger volume of nectar at night (2.83 ± 0.15 μL) than during the day (1.50 ± 0.13 μL). There was no significant difference in sugar concentration (g sucrose per 100 g solution, known as % Brix) between nectar secreted at night (16.51 ± 0.56 %) and that secreted during the day (14.96 ± 0.71 %) (*P* = 0.086, Wald χ^2^ = 2.955, df = 1) (Fig. 2A). Moreover, the L-morph secreted significantly (*P* = 0.038, Wald χ^2^ = 4.302, df = 1) more nectar (3.13 ± 0.23 μL) than the S-morph (2.53 ± 0.16 μL) at night. The nectar volume produced during day by L- (1.65 ± 0.20 μL) and S-morphs (1.35 ± 0.15 μL) did not differ significantly (*P* = 0.234, Wald χ^2^ = 1.419, df = 1). There was no significant difference in the sugar concentration of nectar produced by L- and S-morphs (all *P* > 0.05) (Fig. 2A).

Fructose, glucose and sucrose were detected in *T. sinensis* nectar by high performance liquid chromatography (HPLC) analysis. The sucrose content (9.15 ± 0.65 g/mL) in nectar (all nectar data of L- and S-morphs combined together and analysed) was significantly higher (*P* < 0.001, Wald χ^2^ = 51.145, df = 2) than the fructose (4.75 ± 0.34 g/mL) and glucose contents (5.96 ± 0.37 g/mL). There was no significant difference in nectar sugar composition (the proportions of fructose, glucose and sucrose) between the L- and S-morphs (*P* = 0.212, Wald χ^2^ = 1.556, df = 1). In nectar of the L-morph, the sucrose content was significantly higher than the fructose content, and the glucose content did not differ significantly from the sucrose and fructose contents (*P* = 0.005, Wald χ^2^ = 10.538, df = 2) (Fig. 2B). In the nectar of the S-morph, the sucrose content was significantly higher than the fructose and glucose contents, and the fructose and glucose contents did not differ significantly (*P* < 0.001, Wald χ^2^ = 48.048, df = 2) (Fig. 2B). The nectar of two morphs in *T. sinensis* is rich in sucrose and the sucrose/hexose ratio of S-morph flowers (*r* = 1.10) was higher than that of L-morph flowers (*r* = 0.70).

### Pollinator species and abundance

Hawkmoths (*Macroglossum*), bumblebees (*Bombus*) and honeybees (*Apis*) were the major floral visitors of *T. sinensis* during the field observations in 2018 and 2019. When the long-tongued hawkmoths probed the nectar at the base of the narrow floral tube pollen was transferred onto the tongue. The tongue could touch another stigma and achieve effective pollination (Fig. 1B. i, ii). The bumblebees only robbed the nectar (Fig. 1B. iii), and the honeybees mainly groomed the *T. sinensis* pollen into their corbiculae (Fig. 1B. iv). We found that *Macroglossum* frequently pollinated *T. sinensis* flowers at 18:00–20:30 but seldomly appeared during the rest of the day.

**Fig. 3:**
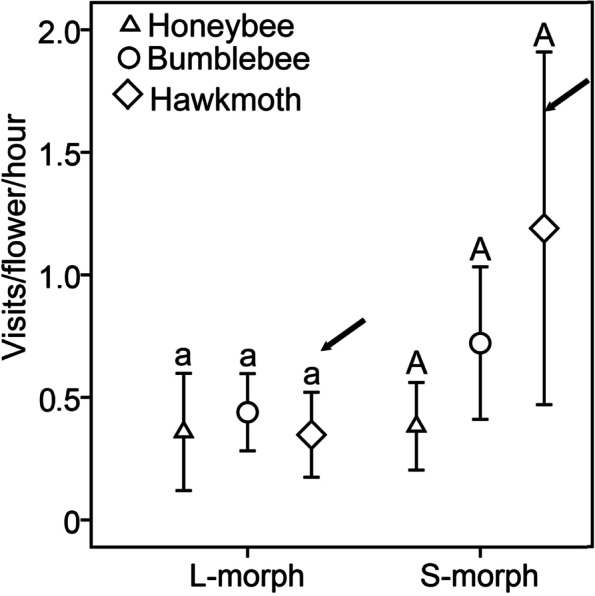
Comparison of visits/flower/hour between honeybees, bumblebees and hawkmoths to S- and L-morphs. Bars sharing the same letters are not significantly different in visit rates among three visitor groups. The visit rates of hawkmoths to the S-morph are significantly higher than those to the L-morph (marked with black arrows)

The visit rates of hawkmoths (0.81 ± 0.20 visits/flower/hour), bumblebees (0.60 ± 0.09 visits/flower/hour) and honeybees (0.37 ± 0.06 visits/flower/hour) to *T. sinensis* were not significantly different (*P* = 0.247, Wald χ^2^ = 2.797, df = 2). The visit rates of these insects to the L- morph (0.86 ± 0.17 visits/flower/hour) were significantly higher (*P* = 0.014, Wald χ^2^ = 6.056, df = 1) than the visit rates to the S-morph (0.38 ± 0.05 visits/flower/hour). There was no interaction between visitor types and floral morphs with respect to visit rate (*P* = 2.125, Wald χ^2^ = 3.072, df = 2). Moreover, the visit rate of hawkmoths to the S-morph (1.19 ± 0.35 visits/flower/hour) was significantly higher (*P* = 0.030, Wald χ^2^ = 4.683, df = 1) than that to the L-morph (0.35 ± 0.08 visits/flower/hour) (Fig. 3).

### Pollen transfer efficiency of hawkmoths

Hawkmoths removed significantly more pollen from the anthers and deposited more pollen grains on the stigmas of L-morph than the S-morph during a single visit (Table 2). Moreover, the number of pollen grains removed per visit, as a proportion of the total number of pollen grains in virgin flowers, was significantly higher in the L-morph (0.87 ± 0.02, *n* = 48) than in the S-morph (0.78 ± 0.03, *n* = 46) (*P* = 0.021, Wald χ^2^ = 5.336, df = 1).

**Table 2:** Comparisons of pollination efficiency (pollen removal and pollen receipt of one visit by a hawkmoth) (mean ± SE, sample size) between the L- and S-morphs of *Tirpitzia sinensis* analyzed by GLM. Values of the L-morph significantly larger than those of the S-morph are written in bold

	L-morph **(48)**	S-morph (46)	Wald *χ*^2^	df	*P*
Pollen removal	**2774.6 ± 73.6**	994.3 ± 37.3	418.602	1	< 0.001
Pollen receipt	**66.4 ± 12.0**	9.9 ± 1.8	51.408	1	< 0.001

### Breeding system of *T. sinensis*

**Fig. 4:**
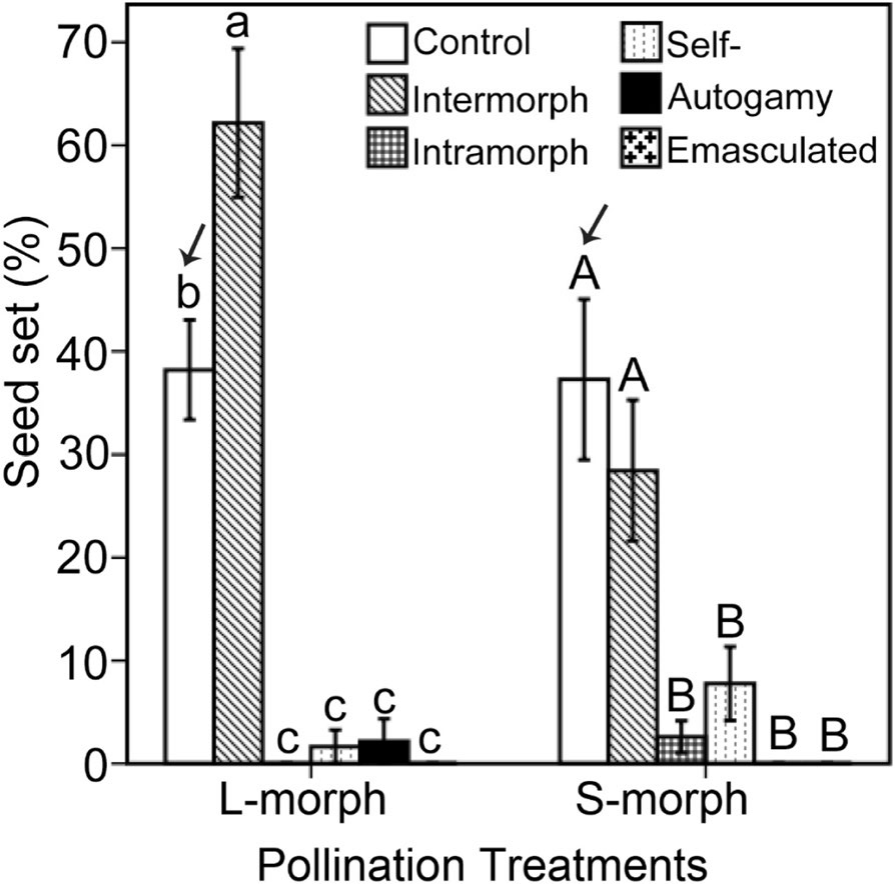
Comparison of seed set under control, intermorph, intramorph, self-, autogamy and emasculated pollination treatments in L- and S-morphs. Bars sharing the same letters are not significantly different in seed set among pollination treatments. The seed set under control treatment between S- and L-morphs had no significant difference (marked with black arrows)

*T. sinensis* had a cryptic self-incompatibility system because nearly no seeds were produced following intramorph pollination (seed set 1.44 ± 0.89 %, *n* = 60; data of L- and S-morph pollination treatments combined together and analysed). By contrast, the seed set under intermorph pollination was high (43.47 ± 5.45 %, *n* = 60). The seed set of L-morphs as pollen recipients (19.16 ± 2.54 %) was not significantly different from that of S-morphs (11.65 ± 2.01 %) (Table 3). For the L-morph, the fruit set of intermorph pollination (62.17 ± 7.23 %, *n* = 30) was significantly higher than that of the open-pollinated control treatment (38.19 ± 4.85%, *n* = 30), intramorph pollination treatment (0.00 ± 0.00 %, *n* = 30), self-pollination treatments (1.63 ± 1.63 %, *n* = 30), autogamy treatments (2.17 ± 2.17 %, *n* = 30) and emasculated treatments (0.00 ± 0.00 %, *n* = 30) (*P* < 0.001, Wald χ^2^ = 234.255, df = 5) (Fig. 4). For the S-morph, the fruit set of intermorph pollination (28.45 ± 6.82 %, *n* = 30) was not significantly different from that of the control treatment (37.27 ± 7.78 %, *n* = 30), but was significantly higher than that of intramorph pollination treatments (2.59 ± 1.57 %, *n* = 30), self-pollination treatment (7.76 ± 3.58 %, *n* = 30), autogamy treatments (0.00 ± 0.00 %, *n* = 30) and emasculated treatments (0.00 ± 0.00 %, *n* = 30) (*P* < 0.001, Wald χ^2^ = 75.451, df = 5) (Fig. 4). Significant interactions were found between pollen recipient morph and pollination treatments with respect to seed set (Table 3). Moreover, under the control treatment there was no significant difference in seed set between L- and S-morphs (*P* = 0.999, Wald χ^2^ = 0.00, df = 1).

**Table 3:** Generalized linear model: effect of pollen recipient morph (L- and S-morphs) and pollination treatments (control, intermorph, intramorph, self-, autogamy and emasculated) and their interaction on seed set (%) in *Tirpitzia sinensis*

Source of variation	df	Wald χ^2^	*P*
Pollen recipient morph	1	0.965	0.326
Pollination treatments	5	238.863	<0.001
Interaction	5	23.962	<0.001

## Discussion

The different morphs of the distylous *T. sinensis* have various adaptive strategies to ensure reproductive success. In this heterostylous plant the floral traits and the sugar concentration, composition and secretion rhythm of nectar are adapted to hawkmoth pollination (*Macroglossum* spp.). Hawkmoth pollination efficiency is promoted in the L-morph by the exserted stigma, numerous pollen grains per anther, and large volume of nectar, and in the S-morph by the high sucrose/hexose ratio in the nectar. The seed set in the field does not differ between the two morphs.

Hawkmoths of the genus *Macroglossum* are major effective pollinators for plants in tropical and temperate areas and usually pollinate white or creamy-white flowers with a tubular corolla, a strong scent, and low nectar sugar concentration [22, 23]. *Habenaria epipactidea* (Orchidaceae) has a long nectar spur and hawkmoths insert their proboscides into the spur and remove pollinaria on their legs [24]. The distylous tree *Palicourea tetragona* (Rubiaceae) is primarily and commonly pollinated by *Eumorpha labruscae* and *Manduca occulta* (Sphingidae) [25]. Due to their exceptionally long proboscis, *Macroglossum* spp. can contact the anther and stigma of the L- and S- morphs of *T. sinensis* with different parts of the proboscis, facilitating disassortative pollination between morphs.

Floral traits reflect adaptation to a certain group of pollinators [26, 27]. The nectar spur of *Angraecum sesquipedale* (Orchidaceae) is 30–40 cm long and it is pollinated by a hawkmoth whose tongue is about 30 cm long [22]. Long-tongued insects are also important pollinators for distylous plants. *Narcissus papyraceus* (Amaryllidaceae) shows style dimorphism and has tubular flowers. When long-tongued insects (butterflies, moths, nectar-feeding bees) probe into the base of the corolla to seek nectar, the pollen of L-morphs and S-morphs could be deposited on the different parts of the proboscis and thus achieve disassortative pollination [28]. The reciprocal herkogamy of *Dunnia sinensis* (Rubiaceae) gives a mechanical fit to the bodies of the pollinators (bees and butterflies). L-morph pollen is mainly deposited on the head and S-morph pollen is deposited on the abdomen, thus achieving intermorph pollination [29].

Flowers pollinated by hawkmoths produce copious nectar to support the large energy requirement of the visitor. The nectar is usually dilute, ensuring that it flows easily through the slender proboscis [30–32]. *Petunia axillaris* (Solanaceae), with a large volume (approximately 35 μL) of nectar of low sugar concentration (approximately 16%) [33], is pollinated by hawkmoths. *Lilium sargentiae* (Liliaceae**)** is pollinated by long-tongued hawkmoths (*Agrius convolvuli*) and secretes a high volume (approximately 15 μL) of dilute (sugar concentration approximately 27%) nectar [34]. Similarly, the nectar sugar concentration of *T. sinensis* is low. Although each flower secretes a small amount of nectar, the plant still has numerous flowers on each inflorescence.

Nectar traits can affect pollinator behaviour. By manipulating the nectar volume of *Mirabilis multiflora*, hawkmoths visited significantly more flowers with larger amounts of nectar [31]. The nectar of hawkmoth-pollinated *Petunia axillaris* is rich in sucrose (approximately 57% of the nectar sugar). The amount of nectar produced by mutational *P. axillaris* individuals per flower is only 1/3 of that produced by wild-type individuals; hawkmoth pollinators reduce the probing duration on low-nectar plants when they are exposed simultaneously to the mutational and wild-type *P. axillaris* [33]. The nectar sugar composition could potentially act as an important influence on visitor activities. Hummingbirds, hawkmoths and long-tongued bees prefer sucrose-rich nectar, and short-tongued bees and flies prefer hexose (glucose and fructose)-rich nectar [15, 35]. *T. sinensis* secreted a greater volume of nectar by night than by day, and the nectar is rich in sucrose. This may be a possible adaptation to pollination by *Macroglossum* flying at dusk and by night.

Numerous studies have focused on the differences between the morphs of distylous plants in flower morphology, pollination and reproductive success. The S-morph of *Fagopyrum esculentum* secretes more nectar with a higher proportion of sucrose than L-morph flowers, and honeybees visit S-morph inflorescences more frequently and spend longer on them [15]. The L-morph of hummingbird-pollinated *Palicourea padifolia* produced higher nectar volumes than S-morph flowers, which caused the L-morph to suffer more herbivore damage than S-morph flowers. The long-styled plants of *P. padifolia* produced significantly less fruit mass than short-styled plants [36]. The S-morph flowers of buckwheat (*Fagopyrum esculentum*) produced larger and fewer pollen grains and secreted more nectar with a higher ratio of sucrose than L-morph flowers. The S-morph flowers were more likely to be visited by honeybees, but fewer pollen grains were deposited on the stigmas at different times during the day. There were no significant differences between the two morphs in the numbers of pollen tubes in the styles, seed set or seed weight [15]. Intermorph pollen loads on the stigmas of L- morph individuals were larger than those on the stigmas of S-morphs, and the fruit set was higher in L- morph (31%) than in S-morph individuals (22%) [37]. The L-morph flowers of *Polygonum criopolitanum* produced significantly smaller but more numerous pollen grains than S-morph flowers, and there was no significant difference between the two morphs in the stigma papillae [38].

Floral characters of the two morphs of *T. sinensis* can be regarded as adaptations to ensure reproductive success. The L-morph of *T. sinensis* produced more numerous but smaller pollen grains than the S-morph. The S-morph secreted significantly less nectar volume at night than the L-morph, and hawkmoths might probe for a shorter time in S-morph flowers than in L-morph flowers. So, the pollination efficiency (pollen receipt/pollen removed per visit of hawkmoths could be lower on S-morph flowers than on L-morph flowers. The nectar sucrose/hexose ratio of S-morph flowers was higher than that of L-morph flowers, so S-morph flowers may be more attractive to hawkmoths. However, there was no significant difference in seed set between the two morphs. Seed set under the open-pollinated control treatment was significantly lower than that under the intermorph pollination treatment in the L-morph, but not significantly in the S-morph. The L-morph of *T. sinensis* may exhibit legal (intermorph) pollen limitation, perhaps due to the low visit rate of hawkmoths.

Since Darwin's hypothesis of promotion of cross pollination, heterostylous plants have been thought to promote compatible pollination between floral morphs within species [1]. To test the hypothesis, legal (intermorph) and illegal (intramorph) pollen must be detected on the stigma of L- and S-morphs. Most heterostylous species have tubular flowers (such as *T. sinensis*), and some are bowl-shaped flowers. However, the reproductive strategies of the two types of corolla formations in heterostylous plants remain unknown. In Linaceae, the tubular flowers of distyly *T. sinensis* are pollinated by hawkmoths (*Macroglossum*), and the bowl-shaped flowers of distyly *Linum suffruticosum* are pollinated by bee-flies (Bombyliidae) [39]. We plan to further explore whether the opening degree of corollas could affect the compatible pollination using *Tirpitzia* and *Linum* species.

## Conclusion

The distylous *T. sinensis* is effectively pollinated by hawkmoths (*Macroglossum*). The visit rate of hawkmoths is higher, and the pollination efficiency is lower, in the S-morph than in the L-morph. Visiting bumblebees always rob the nectar, and honey bees mainly act as pollen thieves. *T. sinensis* is self-incompatible, and there is no significant difference between morphs in natural seed production. The long floral tube and nectar traits (large amount secreted by night, low sugar concentration and high proportion of sucrose) of *T. sinensis* are seen as adaptations to hawkmoth pollination. Moreover, the exserted stigma, large numbers of pollen grains per anther and large volume of nectar of the L-morph could increase the hawkmoths’ pollination effectiveness. L-morph stigmas are usually accessible to pollinators [7, 13, 39]. The higher sucrose/hexose ratio in the nectar of the S-morph might attract hawkmoths to visit the inflorescence.

## Methods

### Study species and sites

We performed all the field experiments in Laoshan Provincial Nature Reserve (104°49′ 62" E, 23°94′ 8" N, approximately 1700 m above sea level), Malipo country, Yunnan province, southwest China.

Plants of *T. sinensis* were obtained under the permission of Laoshan Nature Reserve Bureau, Yunnan province, China. The formal identification of the plant was undertaken by Liu Changqiu, associate researcher, Guangxi Institute of Botany, Chinese Academy of Sciences. A voucher specimen photo of *T. sinensis* has been deposited in Plant Photo Bank of China (PPBC); the deposition number is xyc74220920100731.

### Difference in traits between the L- and S-morphs of *T. sinensis*

To compare plant performance between the two morphs, we randomly chose 50 plants per morph, selected one flower from each plant and measured two vegetative and fourteen reproductive traits, including leaf length and width; sepal length and width; corolla length and width; opening diameter; tube depth and diameter; blade length and width; stamen length; pistil length; and anther length, width and thickness to 0.01 mm using a caliper micrometre.

To compare pollen, ovule production and pollen size, we selected 30 L-morph flower buds and 30 S-morph buds from different individuals and stored them in 1.5-mL centrifuge tubes filled with 75 % alcohol for fixation and preservation. The anther and ovary from one flower bud per plant were separated using forceps in the laboratory, and the anthers were suspended in 500 μL water. In each pollen sample we counted the pollen grains in three 50-μL drops under a Nikon E100 optical microscope. The mean pollen grain number of the three drops was multiplied by 10 to estimate the pollen production of one flower. The ovules were counted under a stereomicroscope. The P/O ratio was equal to the number of pollen grains divided by the corresponding ovule number. For pollen size estimation, mature flowers from different individuals of the L-morph (*n* = 30) and S-morph (*n* = 30) were stored in 75 % alcohol in 1.5-mL centrifuge tubes. In the lab, the pollen grains of each flower were photographed with a fluorescence microscope, and the polar axis length and equatorial axis length of three pollen grains in each flower photo were measured and analysed using Digimizer Version 4.6.0. To compare the flower lifetime in the two morphs, we marked one bud of each from the above selected 30 individuals of L- and S-morphs and recorded the first day of opening. Every two days, we recorded the state of the flower until the anthers and pistil lost function.

### Comparison of nectar volume, sugar concentration and composition between L-morph and S-morph of *T. sinensis*

To compare the nectar volume and concentration in *T. sinensis* during anthesis between day and night, we bagged and labelled 60 flowers before anthesis (one from each of 30 plants of each morph). During the male flowering phase, the nectar in the bagged flower was removed using glass microcapillary tubes (0.3 mm in diameter) on the day before the measurement. Nectar was extracted from the flowers bagged from 18:30 to 06:30 (secreted during the night). After the treatments, the same flower was bagged again, and the nectar was extracted from 06:30 to 18:30 (secreted during the day) the next day. The length (L) of the microcapillary tube occupied by nectar was measured using a caliper micrometre. The volume (V_total)_ and length (L_total_) of one standard microcapillary were calculated, and the volume of nectar (V) is equal to L/L_total_ * V_total_. And the concentration of nectar was measured (as g sugar per 100 g solution) with a hand-held refractometer (Eclipse 0 – 50 %; Bellingham and Stanley Ltd., Basingstoke, United Kingdom; see [40]).

To measure sugar composition, we collected nectar from bagged flowers of the L-morph (one from each of 30 individuals) and the S-morph (one from each of 27 individuals) of *T. sinensis* using microcapillary tubes. After the nectar length was measured using a caliper micrometre, the nectar was spotted onto filter paper and air-dried at room temperature [36]. The spotted filter papers were placed in a 1.5 ml centrifuge tube and stored in the refrigerator at − 20 °C. The sugars were removed by elution with 100 µl of deionised water at room temperature for 24 h. Sugar type (glucose, fructose, sucrose and maltose) was identified, and the relative mass was quantified by High Performance Liquid Chromatography (HPLC, Waters Corporation, Milford, Massachusetts) with a refractive index detector and an Agilent Zorbax carbohydrate analysis column 843, 300 – 908 (Agilent Technologies, Santa Clara, California) under a column temperature of 35 °C. The mobile phase was 80 % acetonitrile, the flow rate was 1 ml/min and the injection volume was 20 µL. Quantities of each sugar in nectar samples were determined by the standards (glucose, fructose, sucrose and maltose) using regression equations (based on response peak areas to standard sugar mass) and were expressed as relative percentage by mass [41]. The sucrose/hexose ratio *(r*) of the L-morph and the S-morph in *T. sinensis* was calculated as the amount of sucrose / (amount of glucose + amount of fructose) [42].

### Pollinator species and abundance

To determine the species of pollinator of *T. sinensis*, we observed all visits of different species over 2018 and 2019 in several populations with three or four individuals including hundreds of flowers. Visitors were observed on L- and S-morphs on 9 sunny days (July 15, 16, 17, 19, 21, 22, 23, 24, 26 and 29) in 2018 and 8 sunny days (June 22, 26, 27, 28, 29, 30 and July 1, 3) in 2019. Each session lasted for 30 min between 7:00 and 22:00 h, and the observations of L- and S-morphs were conducted simultaneously. We randomly selected 10 populations containing both L- and S-morph individuals and completed 40 and 44 sessions in 2018 and 2019, respectively. Visitor moves in one population were recorded to quantify visitation rates to L- and S-morphs. Visit number per foraging bout, visitor species and foraging behaviour were recorded, and the total open flowers in each population were counted. The visit frequency was expressed as the mean number of visits per flower per hour of visitor.

### Pollen transfer efficiency of hawkmoths

To compare the pollination efficiency of hawkmoth between the L- and S-morphs, we estimated the pollen removal and receipt per morph. Male-phase inflorescences (previously unvisited) were bagged until anther dehiscence. The inflorescences were unbagged at about 17:30 when the hawkmoths began to be active. Each inflorescence was allowed a single visit by a hawkmoth. Other visitors could be driven away if they appeared in our visual range. Once the flowers were visited by a hawkmoth, they were picked and stored in a 2-mL centrifuge tube in 75 % alcohol. To estimate pollen removal, we collected 48 visited flowers of the L-morph and 46 visited flowers of the S-morph from different plants with another 48 L-morph buds and 46 S-morph buds as the control. Each flower was stored in a 2-mL centrifuge tube with 75 % alcohol. Pollen removal per flower was calculated from the mean number of pollen grains in unvisited flowers minus the mean number of pollen grains remaining after one visit. To estimate pollen receipt per visit, we removed undehisced anthers from the 48 male-phase flowers of the L-morph and 46 male-phase flowers of the S-morph and bagged these flowers with cotton mesh until they developed into the female phase. The bags were removed and the inflorescences were allowed one visit by a hawkmoth. Stigmas of these visited emasculated flowers were collected and stored in a 1.5-mL centrifuge tube with alcohol. Pollen grains from the anthers and on the stigmas were counted under a light microscope (Nikon E100). The anthers were fully mashed with tweezers to form 0.5 mL of pollen suspension. Three 50-μL drops of each pollen sample were counted, and the mean was multiplied by 10 to estimate pollen production (for undehisced anthers) or pollen remaining per flower after a single visit [43].

### Breeding system

To determine whether *T. sinensis* is self- and intramorph incompatible, we conducted artificial pollination experiments as follows: (1) open pollination as control; (2) intramorph pollination (L-morph as pollen receptor receiving L-morph pollen from other individuals and S-morph as pollen receptor receiving S-morph pollen from other individuals); (3) intermorph pollination (L-morph as pollen receptor receiving S-morph pollen from other individuals and S-morph as pollen receptor receiving L-morph pollen from other individuals); (4) self-pollination (pollen from the flowers of the same individuals); (5) autogamy treatments (the flowers were bagged all the time without any treatments); and (6) emasculated treatments. In 30 individuals each for L- and S-morphs, six flowers were marked with a cotton thread of different colours. Four of the six flowers were emasculated and bagged until they developed into the female phase and then received intramorph, intermorph, self- and emasculated pollination treatments. The remaining two flowers were used as the control and autogamy pollination treatments. Three months after pollination, seeds per flower of the six pollination treatments were collected and counted.

## Data analysis

To assess the differences in plant performance between L- and S-morphs, we compared 16 plant vegetative and reproductive traits, flower lifetimes and pollen/ovule ratio, P/O (pollen grain number/ ovule number) using a generalized linear model (GLM) with normal distribution and identity-link function. The pollen grain number and ovule number were compared between L- and S-morphs using Poisson distribution with loglinear-link function in GLM (all plant characters as dependent variable, and L- and S-morphs as factors). Nectar volume and sugar concentration were analysed using GLM with normal distribution and identity-link function (nectar volume and sugar concentration as dependent variables, and L- and S-morphs and day and night as factors) to compare the nectar traits of the two morphs between day and night. Glucose, fructose, sucrose and maltose contents in nectar were examined using GLM with normal distribution and identity-link function (sugar composition as dependent variables, and L- and S-morphs as factors) to compare the sugar composition between the two morphs. Data of visits were analysed using GLM with normal distribution and identity-link function (visitation rates as dependent variables, and flower morphs and visitor types as factors) to compare the visiting rates (visits/flower/hour) of all visitors between the two morphs. Pollen removal and receipt between the two morphs were compared using GLM with Poisson distribution with loglinear-link function (pollen number as dependent variable, and L- and S-morph as factors). Seed sets of all treatments were examined with binary logistic analysis in GLM (full seed number as event variable, total ovule number as trait variable, and pollination treatments and flower morph as factors) to compare the reproductive success of six pollination treatments between the two morphs. All data were analysed in SPSS 20.0 (IBM Inc., New York, NY) software.

## Data Availability

All data generated or analyzed during this study are included in this published article.

## Abbreviations

*T. sinensis*: *Tirpitzia sinensis*
L-morphs: Long-styled morphs
S-morphs: Short-styled morph
L: The length of the microcapillary tube occupied by nectar
V_total_: The volume of one standard microcapillary
L_total_: The length of one standard microcapillary
V: The volume of nectar
HPLC: High Performance Liquid Chromatography
GLM: Generalized linear model
P/O: Pollen number / ovule number
